# Scoliosis among children in Qinghai-Tibetan Plateau of China: A cross-sectional epidemiological study

**DOI:** 10.3389/fpubh.2022.983095

**Published:** 2022-08-19

**Authors:** Lijin Zhou, Honghao Yang, Yong Hai, Junrui Joanthan Hai, Yunzhong Cheng, Peng Yin, Jincai Yang, Yangpu Zhang, Yunsheng Wang, Yiqi Zhang, Bo Han

**Affiliations:** ^1^Department of Orthopedic Surgery, Beijing Chao-Yang Hospital, Beijing, China; ^2^Princeton International School of Mathematics and Science, Princeton, NJ, United States

**Keywords:** scoliosis, Qinghai-Tibetan Plateau, prevalence, associated factors, Tibetan ethnicity

## Abstract

**Background:**

The average altitude of Qinghai-Tibetan Plateau is 4,500 m and most of the residents are Tibetan ethnicity. The purpose of this study was to investigate the prevalence of scoliosis and associated factors among children in this region through a scoliosis screening program.

**Methods:**

A cross-sectional study was preformed between May 2020 and December 2020 in Qinghai-Tibetan Plateau. A total of 9,856 children aged 6–17 years from schools and nearby villages were screened using visual inspection, the Adams forward-bending test, the angle of trunk rotation, and radiography. A self-designed questionnaire was used to collect demographic data. The prevalence of scoliosis and associated factors were analyzed.

**Results:**

The overall prevalence of scoliosis among children in Qinghai-Tibetan Plateau was 3.69%, with 5.38% for females and 2.11% for males. The prevalence of scoliosis was 3.50% in children resided below 4,500 m while 5.63% in those resided above 4,500 m (*P* = 0.001). The prevalence of congenital scoliosis (2.14 vs. 0.42%, *P* < 0.001) and neuromuscular scoliosis (0.34 vs. 0.07%, *P* = 0.041) were significantly higher in the altitude above 4,500 m. 50.00% of patients resided above 4,500 m were recommended for surgery while 16.24% in those resided below 4,500 m (*P* < 0.001). Independent associated factors were detected as female (OR = 2.217, 95 CI% 1.746–2.814, *P* < 0.001), BMI < 18.5 (OR = 1.767, 95 CI% 1.441–2.430, *P* = 0.005), altitude of residence ≥ 4,500 m (OR = 1.808, 95 CI% 1.325–2.483, *P* = 0.002), and sleep time < 8 h (OR = 2.264, 95 CI% 1.723–2.846, *P* = 0.001).

**Conclusion:**

The prevalence of scoliosis among children in Qinghai-Tibetan Plateau was 3.69%. With increasing altitudes, the prevalence of scoliosis and its major type were different from that at lower altitudes. Female, BMI < 18.5, altitude of residence ≥ 4,500 m, and sleep time < 8 h were independently associated with the prevalence of this disease. Early screening should be carried out before the age of 7 years, especially in the high-altitude, underdeveloped, and rural areas.

## Key messages:

The prevalence of scoliosis are varying from 0.11 to 2.64% among different provinces in Mainland China. Nevertheless, the epidemiological characteristics of scoliosis in Qinghai-Tibetan Plateau is unknown.
This study revealed that the prevalence of scoliosis among Tibetan children in Qinghai-Tibetan Plateau was 3.69%. Female, BMI < 18.5, altitude of residence ≥ 4,500 m, and sleep time < 8 h were independently associated with the prevalence of this disease.
This study indicated that early screening should be carried out before the age of 7 years, especially in the high-altitude, undeveloped, and rural areas.

## Introduction

Scoliosis is a complex three-dimensional deformity of the spine with a coronal curvature more than 10° (1). The etiology of scoliosis is various, mainly including idiopathic, congenital, neuromuscular, and syndromic factors (2). Congenital scoliosis indicates that the spinal deformity arise from vertebral anomalies presenting at birth, such as hemivertebra (3). Neuromuscular scoliosis is caused by primary neuropathies or myopathies, such as cerebral palsy and Duchenne muscular dystrophy (4). Syndromic scoliosis is spinal deformity associated with systemic disease, such as Marfan syndrome, Prader-Willi syndrome, and achondroplasia (5). Idiopathic scoliosis is an exclusive diagnosis, indicating that clear etiology could not be found.

The early stage of scoliosis usually had an asymptomatic nature. However, the progression of untreated scoliosis could result in severe thoracic deformity, profound cardiopulmonary compromise, neurological impairment, and social psychogenic problems (6). Therefore, early detection and intervention of scoliosis through screening program in children is of great importance.

The prevalence of scoliosis in previous international epidemiological studies ranged from 0.20 to 3.10% (7–15). In Mainland China, a meta-analysis by Zhang et al. reported that the prevalence of scoliosis was 1.02% among the primary and middle school students (16). However, due to distinct environmental factors and socioeconomic factors, the prevalence are varying from 0.11 to 2.64% in different provinces (16). Also, the studies included in this mate-analysis mainly investigated the Han population living on plain region. The prevalence of scoliosis differs among races but the data of Tibetan population is still limited.

Qinghai-Tibetan Plateau is one of the highest areas with people living on Earth (17). The average altitude is 4,500 m and most of the residents are Tibetan ethnicity (18). With the increasing altitude, the oxygen concentration in air would decrease rapidly (19). Hypoxic conditions may have effects on the progression of some diseases. Also, due to its remote location, the economic development is slow and the medical resources is limited. Many epidemiological studies performed in this region had found significant differences in prevalence of diseases compared with the plain region (20–23). Nevertheless, the epidemiological characteristics of scoliosis in this region is unknown

The purpose of this study was to investigate the prevalence of scoliosis and associated factors among children in Qinghai-Tibetan Plateau of China through a scoliosis screening program.

## Materials and methods

### Study design and sampling

This cross-sectional study was preformed between May 2020 and December 2020 in south-east regions of Qinghai-Tibetan Plateau (Yushu State, Qinghai Province), consisting of a prospective survey on the prevalence of scoliosis in Tibetan children, and a retrospective analysis on the type of all patients diagnosed with scoliosis based on altitudes of residence (below or above 4,500 m) and presenting ages. According to the level of urbanization, four cities or counties were randomly selected for sampling in this study, including the capital of Yushu State (Yushu City), two medium counties (Zaduo county and Zhiduo county), and a relatively less developed country (Qumalai county). This study was approved by the Disabled Persons' Federation of Yushu State, local Education Ministry, and the Research Ethics Committee of Beijing Chao-Yang Hospital (2020-10-21-3).

### Participants

The Tibetan children aged 6–17 years from primary schools, junior high schools, senior high schools, and nearby villages were participants. A cluster sampling method was applied in this study. For screening in schools, a sequence number (from one) was gave to each class, such as 1, 2, 3, 4….; For screening in nearby villages, a sequence number (from one) was also gave to each family. Children from the classes or families with the odd numbers (e.g., 1, 3, 5, 7…) were included. We excluded the children who had been examined or treated by spine specialists before this study. The children in other ethnic group or whose caregivers refused to participate were also excluded.

### Survey instrument

A self-designed questionnaire with satisfactory reliability and validity in pre-survey was used to collect demographic data of each child participating in this study: age, gender, body mass index (BMI), number of children in family, altitude of residence, location of residence, annual household income, history of myopia, appetite, sleep time, daily exercise time, and cognition of scoliosis. The distance between residence and the nearest qualified medical center <30 km indicates the urban residence, otherwise the rural residence.

### Scoliosis screening program

The screening program included three stages: first in Schools Health Care Institute (SHCI); second in local hospital; and third in Beijing Chao-Yang Hospital (CYHP). The chairman of each school and the director of surrounding village was contacted before the screening program. They provided written consent on behalf of the children involved in this study. The caregivers were informed of the purpose of this study and the details of the examination procedure.

In SHCI, the teachers and children were educated about scoliosis and informed of the study contents. Initial scoliosis screening was performed by trained physicians from SHCI using visual inspection, the Adams forward-bending test, and the angle of trunk rotation (ATR) measured by Bunnell scoliometer. The screening procedure was recommended by the consensus of Scientific Society on Scoliosis Orthopedic and Rehabilitation Treatment (SOSORT) (24). The whole process of screening was supervised by a spine specialist from CYHP. Participated children were visually inspected in the upright position. The spine alignment, head lean, shoulder asymmetries, scapular prominence, breast asymmetries, waist asymmetries, pelvic obliquity, and leg length discrepancy were examined. Then, the children were asked to flex trunk forward, looking down, extending elbows and knees, relaxing shoulders, putting palms in front of the knees and keeping feet 15 cm apart (12, 25). The Adams forward-bending test was performed to observe thorax rotation, scapular asymmetries, pelvic obliquity, and spinous process line, diminishing the impact of non-structural scoliosis. Children with any positive clinical sign were inspected for ATR. Children with an ATR >5 degrees were examined again by the spine specialist from CYHP. If positive signs were confirmed in the rescreening, these children would be referred for standing anteroposterior radiography of the whole spine in local hospital. The Cobb angle and the Risser sign were evaluated by two spine surgeons from CYHP. If the Cobb angle was >10°, the final diagnoses of scoliosis was decided.

Personalized treatment was evaluated by a spine specialist from CYHP, according to the severity of scoliosis, the risk of progression, and skeletal maturity. Patients requiring surgical intervention would be referred to CYHP.

### Data management and statistical analysis

Data obtained from questionnaires were transformed and pooled into a database file for the statistical analysis.

All statistical analyses were performed utilizing SPSS version 25.0 (Chicago, IL, USA). The data were presented as mean ± standard deviation for continuous variables with normal distribution; otherwise the median and interquartile range were used. The counts and percentages were presented for categorical variables. The continuous variables were compared by independent sample *t* tests or nonparametric tests, and the categorical variables were compared using Pearson chi-squared tests or Fisher's exact test. *P* values of < 0.05 were considered statistically significant.

Logistic regression analysis was performed to explore the factors associated with scoliosis. Potential factors were firstly selected from the variables in the self-designed questionnaire using univariate logistic analysis. Those that were significant were included into the multivariate logistic analysis to determine independent associated factors with scoliosis. Odds ratio (OR) and its 95% confidence interval (CI) were presented.

### Participant and public involvement

The participants and the public were not involved in the design, recruitment and conduct of the study. There are no plans to disseminate the study findings to the study participants.

## Results

### Summary of scoliosis screening

**Figure 1:**
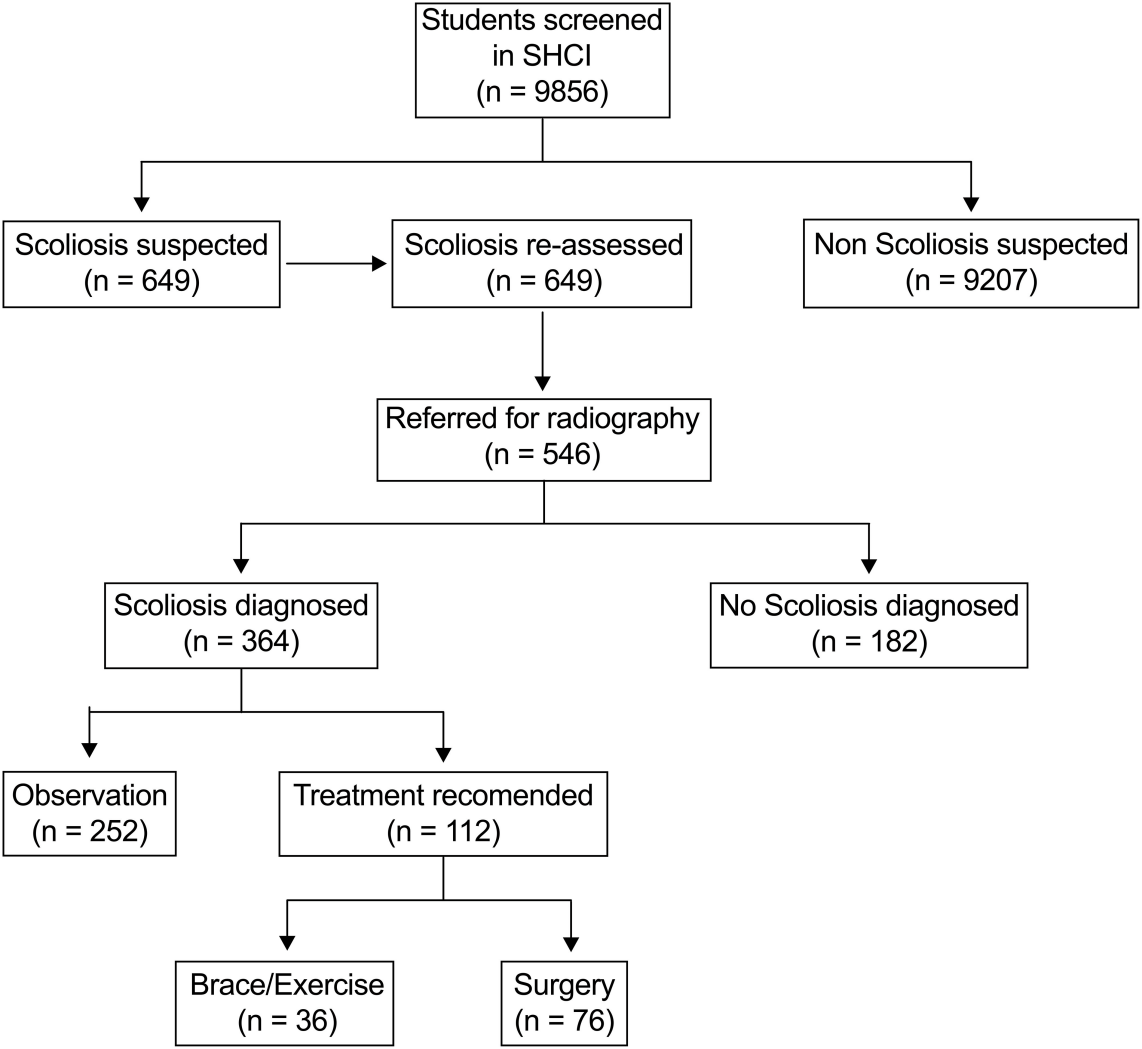
Flowchart of the scoliosis screening program.

A total of 9,856 children aged 6–17 years were screened in this study, including 4,780 females and 5,076 males. In SHCI, 649 (6.58%) children were suspected of scoliosis and re-assessed. 546 (5.54%) children were referred for radiography examinations in local hospital and 364 (3.69%) children were diagnosed as scoliosis. Treatment was recommended for 112 (30.77%) of patients, and 76 (20.88%) of patients need surgical intervention (Figure 1).

### Prevalence of scoliosis by age

**Figure 2:**
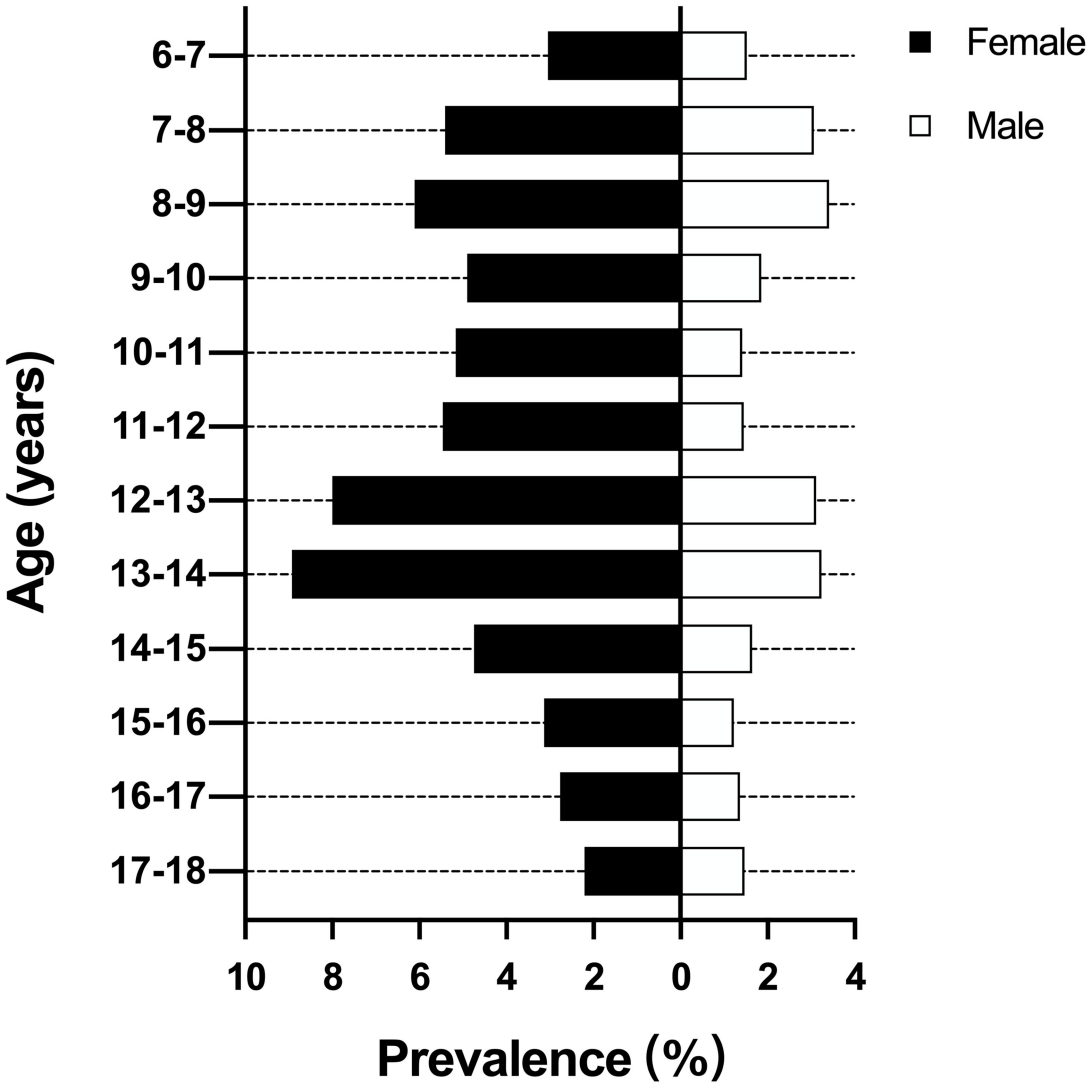
Prevalence of scoliosis according to age and gender.

The overall prevalence of scoliosis was 5.38 and 2.11% in female and male, respectively. The female had a 2.55 times higher prevalence than male. There were two peaks of prevalence at the ages of 8–9 years (4.69%) and 13–14 years (6.05%). Then the prevalence decreased gradually to 1.82% at the ages of 17–18 years (Figure 2). The highest prevalence for female and male were 8.93 and 3.24% at the ages of 13–14 years, respectively.

### Prevalence and type distribution of scoliosis by altitudes of residence

**Figure 3:**
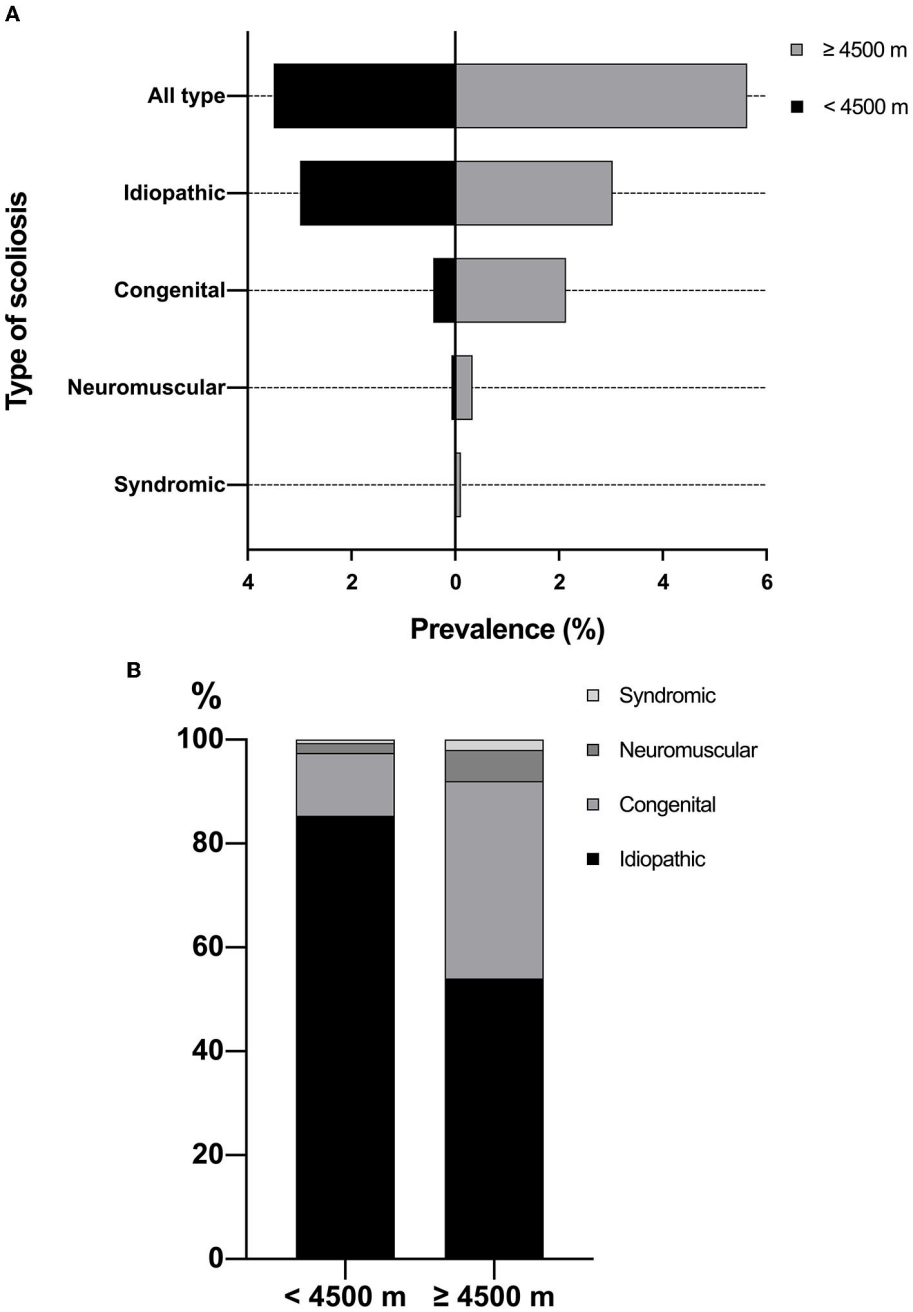
**(A)** Prevalence of scoliosis by altitudes of residence; **(B)** Type distribution of scoliosis by altitudes of residence.

The overall prevalence of scoliosis was 3.50% in children resided below 4,500 m, which was significantly lower than 5.63% in children resided above 4,500 m (*P* = 0.001). In the altitude below 4,500 m, the prevalence of scoliosis was 2.99% for idiopathic scoliosis, 0.42% for congenital scoliosis, 0.07% for neuromuscular scoliosis, and 0.02% for syndromic scoliosis (Table 1 and Figure 3A). The prevalence of congenital scoliosis (2.14 vs. 0.42%, *P* < 0.001) and neuromuscular scoliosis (0.34 vs. 0.07%, *P* = 0.041) were significantly higher in the altitude above 4,500 m. The type distribution of scoliosis was demonstrated in Figure 3B. The proportion of patients needing treatment was significantly greater in the altitude above 4,500 m (58.00 vs. 26.43%, *P* < 0.001). Also, 50.00% of patients resided above 4,500 m were recommended for surgery, which was significantly higher than those resided below 4,500 m (16.24%, *P* < 0.001).

**Table 1:** Prevalence of scoliosis and type by altitudes of residence.

**Prevalence**	<**4,500 m group**			≥**4,500 m group**			*P
	**Total**	**Female**	**Male**	**Total**	**Female**	**Male**	
Idiopathic scoliosis	268/8,968 (2.99%)	199/4,359 (4.57%)	69/4,609 (1.50%)	27/888 (3.04%)	18/421 (4.28%)	9/467 (1.93%)	0.931
Congenital scoliosis	38/8,968 (0.42%)	24/4,359 (0.55%)	14/4,609 (0.30%)	19/888 (2.14%)	9/421 (2.14%)	10/467 (2.14%)	<0.001
Neuromuscular scoliosis	6/8,968 (0.07%)	4/4,359 (0.09%)	2/4,609 (0.04%)	3/888 (0.34%)	1/421 (0.24%)	2/467 (0.43%)	0.041
Syndromic scoliosis	2/8,968 (0.02%)	1/4,359 (0.02%)	$\frac{1}{4}$,609 (0.02%)	1/888 (0.11%)	1/421 (0.24%)	0/467 (0.00%)	0.247
All type of scoliosis	314/8,968 (3.50%)	228/4,359 (5.23%)	86/4,609 (1.87%)	50/888 (5.63%)	29/421 (6.89%)	21/467 (4.50%)	0.001
Treatment recommendation	83/314 (26.43%)	60/228 (26.32%)	23/86 (26.74%)	29/50 (58.00%)	15/29 (51.72%)	14/21 (66.67%)	<0.001
Surgery	51/314 (16.24%)	31/228 (13.60%)	20/86 (23.26%)	25/50 (50.00%)	13/29 (44.83%)	12/21 (57.14%)	<0.001

^*^Indicates the comparison of the total prevalence between groups.

### Factors associated with scoliosis

The demographic characteristics of participants and associated factors with scoliosis were demonstrated in Table 2. Through univariate logistic regression analysis, significant association with scoliosis was found in age (OR = 0.950, 95 CI% 0.917–0.983, *P* = 0.004), female (OR = 2.639, 95 CI% 2.099–3.318, *P* < 0.001), BMI <18.5 (OR = 2.454, 95 CI% 1.940–3.104, *P* < 0.001), altitudes of residence ≥ 4,500 m (OR = 1.644, 95 CI% 1.210–2.235, *P* = 0.001), annual household income <2,000 $ (OR = 6.678, 95 CI% 2.161–21.194, *P* = 0.001), rural residence (OR = 1.962, 95 CI% 1.540–2.500, *P* < 0.001), bad appetite (OR = 3.017, 95 CI% 2.258–4.031, *P* < 0.001), myopia (OR = 0.574, 95 CI% 0.418–0.789, *P* = 0.001), sleep time <8 h (OR = 2.060, 95 CI% 1.595–2.660, *P* < 0.001), and uncognition of scoliosis (OR = 3.192, 95 CI% 1.690–6.029, *P* < 0.001).

**Table 2:** Demographic characteristics of participants and associated factors with scoliosis by univariate logistic regression analysis.

**Variable**	**Scoliosis**	**Non-scoliosis**	**Prevalence %**	**^a^ *P***	**OR**	**95% CI**	**^b^ *P***
**Age**	12.16 ± 2.67	12.63 ± 2.99		0.004	0.950	0.917–0.983	0.004
**Gender**				<0.001			
Male	107 (29.40%)	4,969 (52.35%)	2.11		Ref		
Female	257 (70.60%)	4,523 (47.65%)	5.38		2.639	2.099–3.318	<0.001
**BMI**				<0.001			
≥18.5	98 (26.92%)	4,507 (47.48%)	2.13		Ref		
<18.5	266 (73.08%)	4,985 (52.52%)	5.07		2.454	1.940–3.104	<0.001
**Family children**				0.092			
0	72 (19.78%)	2,278 (24.00%)	3.06		Ref		
1–2	282 (77.47%)	6,863 (72.30%)	3.95		1.300	0.999–1.691	0.051
≥3	10 (2.75%)	351 (3.70%)	2.77		0.901	0.461–1.763	0.762
**Altitudes of residence**				0.001			
<4,500 m	314 (86.26%)	8,654 (91.17%)	3.50		Ref		
≥4,500 m	50 (13.74%)	838 (8.83%)	5.63		1.644	1.210–2.235	0.001
**Annual household income**				<0.001			
≥3,000 $	3 (0.82%)	429 (4.52%)	0.69		Ref		
2,000–3,000 $	69 (18.96%)	2,893 (30.48%)	2.33		3.411	1.069–10.884	0.038
<2,000 $	292 (80.22%)	6,170 (65.00%)	4.52		6.678	2.161–21.194	0.001
**Location of residence**				<0.001			
Urban	271 (74.45%)	8,079 (85.11%)	3.25		Ref		
Rural	93 (25.55%)	1,413 (14.89%)	6.18		1.962	1.540–2.500	<0.001
**Appetite**				<0.001			
Good	213 (58.52%)	7,179 (75.63%)	2.88		Ref		
General	87 (23.90%)	1,598 (16.83%)	5.16		1.835	1.422–2.368	<0.001
Bad	64 (17.58%)	715 (7.53%)	8.22		3.017	2.258–4.031	<0.001
**Myopia**				0.002			
No	180 (49.45%)	4,167 (43.90%)	4.14		Ref		
Unclear	134 (36.81%)	3,310 (34.87%)	3.89		0.937	0.746–1.177	0.577
Yes	50 (13.74%)	2,015 (21.23%)	2.42		0.574	0.418–0.789	0.001
**Sleep time**				<0.001			
≥8 h	284 (78.02%)	8,350 (87.97%)	3.29		Ref		
<8 h	80 (21.98%)	1,142 (12.03%)	6.55		2.060	1.595–2.660	0.040
**Daily exercise time**				0.057			
≥1 h	328 (90.11%)	8,805 (92.76%)	3.59		Ref		
<1 h	36 (9.89%)	687 (7.24%)	4.98		1.407	0.989–2.002	0.058
**Cognition of scoliosis**				<0.001			
Known	10 (2.75%)	750 (7.90%)	1.32		Ref		
Have heard	90 (24.73%)	2,539 (26.75%)	3.42		2.659	1.376–5.135	0.004
Never known	264 (72.53%)	6,203 (65.35%)	4.08		3.192	1.690–6.029	<0.001

^a^*Indicates the results of chi-squared test; ^b^indicates the results of univariate logistic regression analysis*.

In multivariate logistic regression analysis, female (OR = 2.217, 95 CI% 1.746–2.814, *P* < 0.001), BMI < 18.5 (OR = 1.767, 95 CI% 1.441–2.430, *P* = 0.005), altitude of residence ≥ 4,500 m (OR = 1.808, 95 CI% 1.325–2.483, *P* = 0.002), and sleep time < 8 h (OR = 2.264, 95 CI% 1.723–2.846, *P* = 0.001) were detected as the independent associated factors with this disease (Table 3).

**Table 3:** Associated factors with scoliosis by multivariate logistic regression analysis.

	**OR**	**95% CI**	***P***
**Age**	0.912	0.758–1.107	0.125
**Gender**
Male	Ref		
Female	2.217	1.746–2.814	<0.001
**BMI**
≥18.5	Ref		
<18.5	1.767	1.441–2.430	0.005
**Altitudes of residence**
<4,500 m	Ref		
≥4,500 m	1.808	1.325–2.483	0.002
**Annual household income**
≥3,000	Ref		
2,000–3,000	2.257	0.729–12.824	0.264
<2,000	4.382	1.747–20.360	0.101
**Location of residence**
Urban	Ref		
Rural	1.109	0.106–3.384	0.083
**Appetite**
Good	Ref		
General	2.326	0.328–6.002	0.497
Bad	3.815	1.189–7.610	0.084
**Myopia**
No	Ref		
Unclear	1.545	0.361–2.833	0.682
Yes	0.734	0.231–1.569	0.245
**Sleep time**
≥8 h	Ref		
<8 h	2.264	1.723–2.846	0.001
**Cognition of scoliosis**
Known	Ref		
Have heard	2.330	1.069–6.754	0.368
Never known	2.896	1.164–7.045	0.143

## Discussion

This was the first epidemiological study to investigate the prevalence of scoliosis and associated factors in Qinghai-Tibetan plateau of China. The prevalence of scoliosis among children aged 6–17 years was 3.69%, which was higher than Japan (0.87%) (11), Singapore (0.59%) (10), Turkey (2.30%) (13), India (0.61%) (15), Greece (1.70%) (8), Brazil (1.50%) (12), the United States (0.20%) (14), Australia (3.10%) (7), and Nigeria (1.20%) (9). Focusing on Mainland China, the prevalence of scoliosis in high-altitude region was also higher than other plain regions (0.11–2.64%) (16). In addition to genetic variance between Han and Tibetan population, the difference in environment, economic condition, and healthcare access between plateau and plain regions might also contribute to the difference in prevalence of scoliosis.

### Prevalence of scoliosis in Qinghai-Tibetan plateau

Consistent with previous studies, the prevalence of scoliosis began to gradually increase from the ages of 10–11 years (3.24%) and peaked at the ages of 13–14 years (6.05%) (11, 12, 26). However, another peak of prevalence at the ages of 8–9 years (4.69%) was observed among children in our study, which may be the unique clinical features in growth spurt phase. Another potential explanation was the economic backwardness and limited medical resources in this region. In the current study, we found that the annual household income (<2,000 $, OR = 6.678, 95 CI% 2.161–21.194, *P* = 0.001) and location of residence (rural, OR = 1.962, 95 CI% 1.540–2.500, *P* < 0.001) were associated with the prevalence of scoliosis. The prevalence of scoliosis was 4.52 and 6.18% for children in low income and rural residence family, respectively. Although the body abnormality could be observed by their caregivers, the nearby hospitals were not adequate to provide appropriate diagnosis and treatment, and most of their family cannot afford the surgery. Therefore, earlier screening should be carried out before the age of 7 years, especially in the underdeveloped and rural areas.

### Independent associated factors with scoliosis

An important finding in this study was that the both the prevalence of scoliosis and surgery rate were independently associated with the altitudes of residence. For children resided below 4,500 m, the prevalence of scoliosis was 3.50 while 5.63% in those resided above 4,500 m. When focusing on the type, there was no significant difference in the prevalence of idiopathic scoliosis between groups (2.99 vs. 3.04%, *P* = 0.931). Therefore, the higher prevalence in the high-altitude region was mainly due to the congenital (2.14 vs. 0.42%) and neuromuscular scoliosis (0.34 vs. 0.07%). This finding may be owing to the hypoxic conditions in high-altitude region, which could disrupt embryonic development, and then result in organ defects in many systems (27). The development of musculoskeletal and nervous systems could also be impacted by hypoxia, leading to the high prevalence of congenital and neuromuscular scoliosis in the current study. Our previous study revealed that the proportion of mixed vertebral body abnormality, rib anomalies, and intra-spinal malformations was significantly higher in congenital scoliosis patients from Qinghai-Tibetan plateau (28). These patients were commonly complex cases and need undergo surgery, which was consistent with the higher surgery rate (50.00 vs. 16.24%) in this study.

Other associated factors with scoliosis were detected in this study. In agreement with other races in previous studies, females are also more susceptible to scoliosis than males. In all ages, the prevalence of scoliosis in female was higher than male. The female/male ratio in the current study was 2.55:1, which was consistent with the range reported by literature (2.1:1–4.6:1) (8, 10, 29, 30).

BMI was a comprehensive index of reflecting the body shape and nutritional status (31). Ramírez et al. reported a tendency of the patient with scoliosis to have a lower BMI (32). In the present study, the prevalence of scoliosis in patients with BMI < 18.5 was 5.07%, 2.38-fold to those with BMI ≥ 18.5, which was similar to results of a scoliosis epidemiological study in Guangdong Province of China (33). We considered that the low BMI in children with scoliosis was also associated with their inferior appetite. 17.58% of patients reported a bad appetite while it was only 7.53% in children without scoliosis.

Scoliosis could impairs respiratory function by limiting the volume of thoracic cage and the normal inflation of lungs (34). Patients with scoliosis are more susceptible to respiratory events of hypopnea during sleep (35). With the hypoxia conditions in high-altitude region, the sleep breathing of patients with scoliosis may be easily impacted, then reducing the actual sleep time. In this study, the prevalence of scoliosis was higher in children with sleep time <8 h than those ≥ 8 h (6.55 vs. 3.29%, *P* < 0.001), which suggested that the inferior sleep time was independently associated with the prevalence of scoliosis (OR = 2.264, 95 CI% 1.723–2.846, *P* = 0.001).

### Meaningful findings by univariate analysis

Cai et al. reported that myopia was positively associated with scoliosis (31). However, the current study obtained a contrary result (OR = 0.574, 95 CI% 0.418–0.789, *P* = 0.001). As a large proportion of children with scoliosis came from the low income and rural residence family, they usually would not like to accept education in school and had a very low screen time, which were the two strong risk factors of myopia. Therefore, in Qinghai-Tibetan plateau of China, the prevalence of scoliosis was higher in children without myopia than those with myopia (4.14 vs. 2.42%, *P* = 0.002).

For children, caregivers, and teachers, having good cognition of scoliosis is of paramount importance to realize this disease at its early stage and visit the specialists for corrective measures in time. In this study, 72.53% of patients had never known scoliosis while it decreases to 65.35% in healthy children. Therefore, the education of scoliosis to the physicians of SHCI and families having juvenile and adolescent is essential, and more scoliosis screening program in Qinghai-Tibetan plateau of China is indeed.

## Limitation

This study has several limitations. First, the associated factors and scoliosis were not in a cause-and-effect relationship but these factors could help screeners to identify the high-risk population and pay more attention to them. Second, the prevalence of scoliosis was only investigated for 1 year (2020) but the annual prevalence has be variability from year to year. Third, the Cobb angle, curve type, and curve side were not recorded by our investigators, therefore, it was not available to evaluate the curve morphology distribution. Also, due to the original design of this study, the children with scoliosis were not included and their information was not collected. Therefore, the prevalence we reported in this study indicated screening accuracy or the prevalence detected by screening. Last, some answers of variables in the self-designed questionnaire may be unbalanced. We would improve our study design and questionnaire, applying it in subsequent scoliosis screening program

## Conclusion

The prevalence of scoliosis among children in Qinghai-Tibetan plateau of China was 3.69%. With increasing altitudes, the prevalence of scoliosis and its major type were different from that at lower altitudes. Female, BMI < 18.5, altitude of residence ≥ 4,500 m, and sleep time <8 h were independently associated with the prevalence of this disease. Early screening should be carried out before the age of 7 years, especially in the high-altitude, underdeveloped, and rural areas.

## Data availability statement

The raw data supporting the conclusions of this article will be made available by the authors, without undue reservation.

## Ethics statement

The studies involving human participants were reviewed and approved by Disabled Persons' Federation of Yushu State Education Ministry of Yushu State the Research Ethics Committee of Beijing Chao-Yang Hospital. Written informed consent to participate in this study was provided by the participants' legal guardian/next of kin.

## Author contributions

The first draft of the paper was written by LZ and HY. Data calculation and analysis were performed by JH, YW, and YZ. The work was critically revised by YH and LZ. All authors commented on previous versions of the paper, as well as, read and approved the final version. All authors contributed to the research conception and design.

## Conflict of interest

The authors declare that the research was conducted in the absence of any commercial or financial relationships that could be construed as a potential conflict of interest.

## Publisher's note

All claims expressed in this article are solely those of the authors and do not necessarily represent those of their affiliated organizations, or those of the publisher, the editors and the reviewers. Any product that may be evaluated in this article, or claim that may be made by its manufacturer, is not guaranteed or endorsed by the publisher.
